# Notes on the Effect of Injuries to Different Parts of the Cranium

**Published:** 1895-06-29

**Authors:** Alexander Miles

**Affiliations:** Surgical Tutor Royal Infirmary, Edinburgh


### Medical Progress and Hospital Clinics.

[*The Editor will be glad to receive offers of co-operation and contributions from members of the profession. All letters should be addressed to* The Editor, The Lodge, Porchester Square, London, W.]

### NOTES ON THE EFFECTS OF INJURIES TO DIFFERENT PARTS OF THE CRANIUM.

By Alexander Miles, M.D., F.R.C.S.Edin., Surgical Tutor Royal Infirmary, Edinburgh.

There are no two opinions as to the enormous aid which the discoveries regarding the localisation of cerebral function have been to the surgeon, in deciding the appropriate operation for the relief of compression in many cases of head injury. At the same time, not a few cases present themselves in which the extreme degree of compression of the brain produces such conditions that all localising symptoms are obliterated in the general unconsciousness of the patient. In such cases, while the possibility of doing good by operation is present to the mind of the surgeon, he holds his hand in the absence of any clear indication as to where to open the skull. If the operation does no good it may do harm, and in many cases the patient is left to his fate. It is true that in some cases an operation has been performed, and although little or nothing has been found, the symptoms have been relieved. It is equally true that grave cases have recovered without operation. We are not yet in a position to advocate the opening of the skull in all those serious cases for the relief of tension, but it seems probable that this is one of the directions in which cerebral surgery will some day advance.

We must rather try to make use of what evidence, other than localisation signs, we have. The force, direction, and seat of the injury, if properly investigated, may give a clue to the locality in which an operation may be performed with advantage. The writer has made a series of observations on the effects of blows of different intensity over various parts of the skull, a brief résumé of which it is proposed to give.

In investigating clinical cases it is of great value to get the history of the accident from one or more eyewitnesses. This should be done at once, while the vivid impression which such a sight makes on the mind of a person lasts, and before theories have been formed, and discussions held by the witnesses.

External marks of bruising must be observed, and various obvious fallacies in their interpretation guarded against.

In considering the question of the relationship between intra-cranial lesions and the seat of impact on the skull, the question of injury by *contre-coup* inevitably arises. The view that lesions of the brain caused by counter-stroke (*contre-coup*) properly so-called, can take place, has long since been given up by all who have carefully considered the question. The physical conditions and the anatomical arrangement of parts preclude all possibility of such a thing. At the same time it is matter of everyday observation that injuries of the brain, the membranes, and even of the skull itself do occur at the opposite pole of the axis of percussion of a blow. Without going into detail, which has been done elsewhere,* it seems most probable that the physical explanation of this is to be found in the profound disturbance which takes place in the cerebro-spinal fluid, as a result of the momentary depression of the skull at the time of the injury. An analysis of my observations, experimental and clinical, would seem to lend support to the view that there is a definite relationship between the region of the skull struck and the situation of the resulting intra-cranial lesions.

* Miles. Brain—1892, and Lab. Rep. Royal Coll. Physicians, Edin. Vol. iv.

1. **Blows on the Front of the Head.**—Careful observation during life, and post-mortem examination in seven cases in which the blow had been received over the frontal region of the skull, led to the following conclusions: 1. That the severity of the symptoms was in direct proportion to the intensity of the force producing the injury. 2. That frontal blows were more uniformly and more rapidly fatal than blows of equal force received on other parts of the skull. 3. That the lesions at the seat of impact were slight in comparison with those at the opposite pole of the axis of percussion—the so-called point of *contre-coup*. 4. That the opposite pole, being the region of the posterior fossa of the base of the skull, the severe symptoms and the rapidly fatal results were due to hæmorrhages involving the important parts of the brain which lie there. 5. That lesions of the pons, medulla, and fourth ventricle were practically constant.

The following case presents some interesting points: John B., æt. 8, an imbecile, was seen on September 4th, 1889. He had suffered from infantile paralysis when an infant, was quite helpless, and fell like a log if unsupported. Twelve hours before seen he had fallen forward on the hearth-stone, striking his forehead. He became "stiff," unconscious, and on being put to bed, mother said, "went to sleep, and slept all forenoon." Great respiratory difficulty, restlessness, and twitchings of all limbs. Persistent hiccough. When first seen he could not be roused; his breathing was rapid and shallow. Moist sounds all over both lungs. Pulse uncountable, very irregular, and intermittent. Temperature, 103 deg. Bruise size of sixpenny-piece over middle of forehead. Later, the breathing assumed an a-typical Cheyne-Stokes character. There were no localising motor symptoms. He died twenty-four hours after injury. On post-mortem examination, no fracture of skull was found; no cortical lesion. Over upper and anterior aspects of pons and crura cerebri was a thin arachnopial extravasation; various other small effusions in region of base. The fourth ventricle was filled by a large extravasation, and, on removing this, the floor was found studded over with small cayenne-pepper-like spots under the ependyma. Remarks: In a general way this case illustrates the conclusions above stated. The points of special interest are (*a*) the serious lesions in the region of the fourth ventricle; (*b*) the respiratory and cardiac phenomena; (*c*) the comparatively long time he lived after injury.

II. **Blows on the Vertex.**—The axis of percussion in blows over the vertex passes towards the base of the skull, somewhat further forward than in frontal blows. In my cases, the general results of such injuries closely resembled those just described as following on frontal blows.

**III. Blows on Side of Head.**—Eight cases were investigated in which the injury had been sustained on the tempero-parietal region of the head. It was found (1) that although intra-cranial lesions were more diffuse, they were less intense than in frontal or vertex cases: (2) that in almost all cases lesions were found both at the seat of impact and at the opposite pole; if there was any difference of degree at the two points, the lesions at the opposite pole, taken all over, were slightly the more severe; (3) lesions in the region of the bulb were trivial when they existed at all, and the patients lived for some considerable time, in marked contrast to the cases where the injury was frontal; (4) in a large proportion of the cases, evidence of cerebral irritation, in the form of shouting, constant talking, restlessness, and violence, were marked features. The following case illustrates most of these points: T. O., æt. 27, carver in stone, fell from a height of 35 feet on August 14th, 1889, striking the right side of his head on a stone wall as he fell. He landed doubled up on his back. On admission to the Royal Infirmary he was found to have sustained a fracture of the spine in the region of the ninth dorsal vertebra, in addition to head injuries. He was exceedingly noisy and restless, constantly talking, and performing movements with his hands such as are made by a sculptor at work. He gradually became weaker, and died 10 days 18 hours after injury. On post-mortem examination it was found that the spinal cord was completely torn across by a fracture dislocation of the ninth dorsal vertebra. Both cerebral lobes showed slight extravasations, especially the left. Small hæmorrhage on left side of pons. There was no fracture of the skull.

**IV. Blows on Back of Head.**—Nine cases were observed in which the injury was in the occipital region. (1) Although fatal, death followed less rapidly than in frontal cases. (2) The most constant, and necessarily most serious, lesions were found in the region of the pons and medulla, into which structures hæmorrhages were frequent. It is interesting to note that in spite of extravasations into these important parts patients lived from 13 to 37 hours after injury. (3) At the point of *contre-coup* (so-called) the lesions, when present, were of minor importance. (4) The ventricles of the brain sustained less damage than in frontal blows. For example we may take the following case: J. S., æt. 22, received a blow on the occiput by falling into the hold of a ship. Soon after had a "fit." When seen he was unconscious, with left-sided conjugate deviation, paralysis of limbs, several epileptiform attacks, pupils dilated and contracted rhythmically. Regained consciousness 10 hours after. Suddenly his respiration became laboured, and he died 37 hours after injury, remaining conscious to the last. Post-mortem examination revealed a fracture of the occipital bone, passing into foramen magnum, and another in great wing of left sphenoid effusions all over cerebral hemispheres, more especially posteriorly; cerebellum much lacerated. In substance of pons, about upper angle of fourth ventricle, near middle line, were two or three hæmorrhages about the size of large pin-heads. Some blood spots in floor of fourth ventricle.

It would thus appear that by carefully inquiring into the nature of the force, its direction, and the seat of injury to the skull, some indication, at least, may be gained as to the probable seat of the most serious intra-cranial lesions. In all severe head injuries more or less damage is done to parts in the posterior fossa of the skull, a point which seriously affects the prognosis. These lesions are most marked in frontal blows, and least so in lateral ones, hence trephining for the relief of tension offers most hope in the latter case. It is obviously impossible to lay down rules with regard to this point, but no effort must be spared to gain information which may enable us to afford relief in these most distressing cases.

